# Modified Anterolateral Minimally Invasive Surgery (ALMIS) for Total Hip Replacement: Anatomical Considerations, Range of Motion and Clinical Outcomes

**DOI:** 10.3390/medicina59091520

**Published:** 2023-08-23

**Authors:** Christos Koutserimpas, Maria Piagkou, Ilias Karaiskos, Athanasios Karamitros, Konstantinos Raptis, Konstantinos Kourelis, Nikolaos Christodoulou

**Affiliations:** 1 Department of Orthopedics and Traumatology, “251” Hellenic Air Force General Hospital of Athens, Kanellopoulou Av, 11525 Athens, Greece; ilkaraiskos@hotmail.com (I.K.);; 2 Department of Anatomy, School of Medicine, National and Kapodistrian University of Athens, 75 Mikras Asias Str., Goudi, 11527 Athens, Greece; 3 Department of Orthopedics, Athens Medical Group, Psychicko, 11525 Athens, Greece

**Keywords:** hip surgical anatomy, surgical approach, total hip arthroplasty, hip surgery, hip reconstruction, minimally invasive surgery

## Abstract

*Background and Objectives:* In the modified anterolateral minimally invasive surgery (ALMIS) for total hip arthroplasty (THA), the intermuscular plane between the tensor fasciae latae and the gluteus maximus (GM) is exposed, while the anterior ¼ of the GM is detached. There are scarce data regarding this surgical approach. The purpose of the present study is to thoroughly describe this approach, encompassing the anatomical background, and to present the results of a retrospective two-center study of 603 patients. *Materials and Methods:* The present study includes a two-center retrospective observational cohort of 603 patients undergoing the ALMIS technique with minimum 5-year follow-up. Demographics were recorded, while range of motion (ROM) of the hip joint and the Harris Hip Score (HHS) were evaluated preoperatively, at 1, 3 and 12 months postoperatively and at the final follow-up (>5 years). Surgery-related complications were also recorded. *Results:* The studied population’s mean age was 69.4 years, while most of them were females (397; 65.8%). The mean follow-up was 6.9 years. The median HHS at the 1-month follow-up was 74, compared to the 47 preoperatively (*p*-value < 0.0001). At the final follow-up, median HHS was 94. At the 1-month follow-up, mean adduction was 19.9° (compared to 15.4° preoperatively; *p* < 0.0001), mean abduction 24.3° (18.2° preoperatively; *p* < 0.0001), mean flexion 107.8° (79.1° preoperatively; *p* < 0.0001), mean external rotation 20.1° (12.1° preoperatively; *p* < 0.0001) and mean internal rotation 15.3° (7.2° preoperatively; *p* < 0.0001). ROM further improved until the final follow-up; mean adduction reached 22°, mean abduction 27.1°, mean flexion 119.8°, mean external rotation 24.4° and mean internal rotation 19.7°. Regarding complications, 1.3% of the sample suffered anterior traumatic dislocation, in 1.8% an intraoperative femoral fracture occurred, while 1.2% suffered periprosthetic joint infection. *Conclusions:* The modified ALMIS technique exhibited excellent clinical outcomes at short-, mid- and long-term follow-up, by significantly improving hip ROM and the HHS. Careful utilization of this technique, after adequate training, should yield favorable outcomes, while minimal major complications should be expected.

## 1. Introduction

Reconstruction surgery has evolved throughout the years, encompassing minimally invasive techniques, transfusion and perioperative infection reduction protocols, navigation and robotic systems and fast track rehabilitation protocols, aiming to achieve faster recovery and better short- and long-term functional outcomes [1,2,3,4,5].

Total hip arthroplasty (THA) has been considered the “operation of the century”, since it revolutionized the treatment of elderly individuals crippled with osteoarthritis [5]. THA aims to improve the patient’s quality of life and offers good long-term results, by restoring the biomechanical functional characteristics of the hip joint [1,5].

Minimally invasive approaches have been described and studied throughout the last 2 decades [6]. Minimally invasive surgery (MIS) is defined as a surgical technique performed through a short skin incision to avoid injury to muscles and tendons. The advantages of MIS over the classic technique in THA include: faster recovery, shorter rehabilitation and hospital stay, decreased blood loss, less pain and a shorter scar. However, there is still debate regarding the most efficient approach [5,6].

The anterolateral minimally invasive surgery (ALMIS) for THA was initially described in 2004 by Röttinger and, later on, in 2017, modified by Christodoulou [7,8,9]. Röttinger introduced the “anterolateral” minimally invasive technique for total hip replacement, which involved employing the standard Watson-Jones interval. However, a distinct intermuscular plane between the tensor fasciae latae (TFL) and the gluteus maximus (GM) was created without any muscle or tendon incisions or detachment [7,8]. In the modified ALMIS approach, the anterior ¼ of the GM is retracted and the patient is positioned differently from the initially described technique [9,10].

The aim of the present study is to thoroughly describe the modified ALMIS approach, encompassing the anatomical background and possible pitfalls, and to present the results of a retrospective two-center study of 603 patients with minimum 5-year follow-up undergoing this procedure. The retrospective nature of the study allows for longer follow-up which is essential when evaluating patients undergoing THA.

## 2. Materials and Methods

The present study is a retrospective observational 2-center study of prospectively maintained databases. Eligible patients for this study were those suffering hip osteoarthritis and undergoing THA with the ALMIS approach at the Orthopaedics and Traumatology Department of the “251” Hellenic Air Force General Hospital in Athens, Greece and the Department of Orthopedics, Athens Medical Group, Psychicko. Only primary cases were included. The study period was from January 2014 to December 2017 (5 years). Patients had to be re-evaluated for a final >5 years of follow-up to be included in the study.

In all these cases, a threaded cup (EcoFit^®^ SC cup ^®^, Implantcast, Buxtehude, Germany) was placed combined with a curved press-fit stem (EcoFit^®^, Implantcast, Buxtehude, Germany). The cases in which a cemented component was used were excluded from this study. Additionally, patients with neurological or musculoskeletal diseases that could impact functional outcomes were also excluded.

Demographics, including age, gender and body mass index (BMI), were gathered from the medical records. Furthermore, the American Society of Anesthesiologists (ASA) physical status score was also recorded. More particular, the ASA score evaluates the patient’s preanesthesia medical co-morbidities; ASA 1: A normal healthy patient, ASA 2: A patient with mild systemic disease, ASA 3: A patient with a severe systemic disease that is not life-threatening, ASA 4: A patient with a severe systemic disease that is a constant threat to life, ASA 5: A moribund patient who is not expected to survive without the operation and ASA 6: A brain-dead patient whose organs are being removed with the intention of transplanting them into another patient.

The length of in-hospital stay (LHS), from the medical records and the incision scar, at the final follow-up, were also studied.

Range of hip motion and the Harris Hip Score (HHS) were recorded preoperatively and 1 month, 3 months and 12 months postoperatively (comparisons were made with the preoperative value). All patients were re-evaluated for the purposes of this study at final follow-up (minimum 5 years).

Range of hip motion included adduction, abduction, flexion, external rotation and internal rotation and was recorded at these time intervals and compared to the baseline (preoperative) value. All measurements were performed for active movements. In particular, by utilizing a 360-degree universal goniometer, the assessment of hip range of motion was conducted. The patients assumed specific positions: supine for flexion, abduction and adduction measurements; prone for extension measurement; and a seated posture for internal and external rotation measurements. Prior to measurement, subjects were instructed in the required movements. Each measurement was iterated thrice, and the average was documented for both the treated and non-treated sides. For flexion and extension, the goniometer’s pivot point was positioned at the femur’s trochanter major. The immobile arm was aligned with the spinal column, while the mobile arm traced the lateral midline of the femur. During extension measurement, adjustments were made for pelvic elevation and lordosis angle. In the case of abduction and adduction, the goniometer’s pivot point was situated at the trochanter major’s projection on the front of the femur. The stable arm remained parallel to the anterior superior spine of the ilium, and the movable arm followed the anterior midline of the femur. Internal and external rotation assessments were performed with individuals seated, allowing their knees to dangle. The goniometer’s pivot point was established at the tibial tuberosity. The stationary arm was aligned parallel to the ground, and the movable arm tracked the tibial crest. Throughout the measurement process, care was taken to prevent inadvertent hip movements in flexion, extension, abduction and adduction.

The HHS is a clinical assessment tool, evaluating the functional outcomes and overall success of hip surgeries. It measures the patient’s hip function and pain levels before and after the operation. The score ranges from 0 to 100, with higher values indicating better hip function and less pain [11].

Complications, including revision arthroplasty, deep surgical site infection requiring surgery and hip dislocation requiring open or closed reduction, were also evaluated from the medical records and at the final follow-up.

The ethical principles guiding this study were based on the “Declaration of Helsinki, Ethical Principles for Medical Research Involving Human Subjects” (October 2008). The study was conducted in adherence to the guidelines outlined in EN ISO 14155 (Clinical investigations of medical devices for human subjects—Good clinical practices), as well as European and national regulations. Furthermore, the study was approved by the bioethics committee of both institutions (approval number of the “251” Hellenic Air Force Hospital: Φ.400/11/406168/Σ.374/8 February 2021, approval number of the Athens Medical Group, Psychicko Clinic: s23/26 March 2020).

### 2.1. Statistics

Comparison of the quantitative variables between the follow-up time-points were evaluated by Wilcoxon tests. A *p*-value less than 0.05 was considered statistically significant. Statistical analysis was performed using IBM SPSS Statistics version 28.0.

### 2.2. Surgical Technique and Anatomy

**Figure 1:**
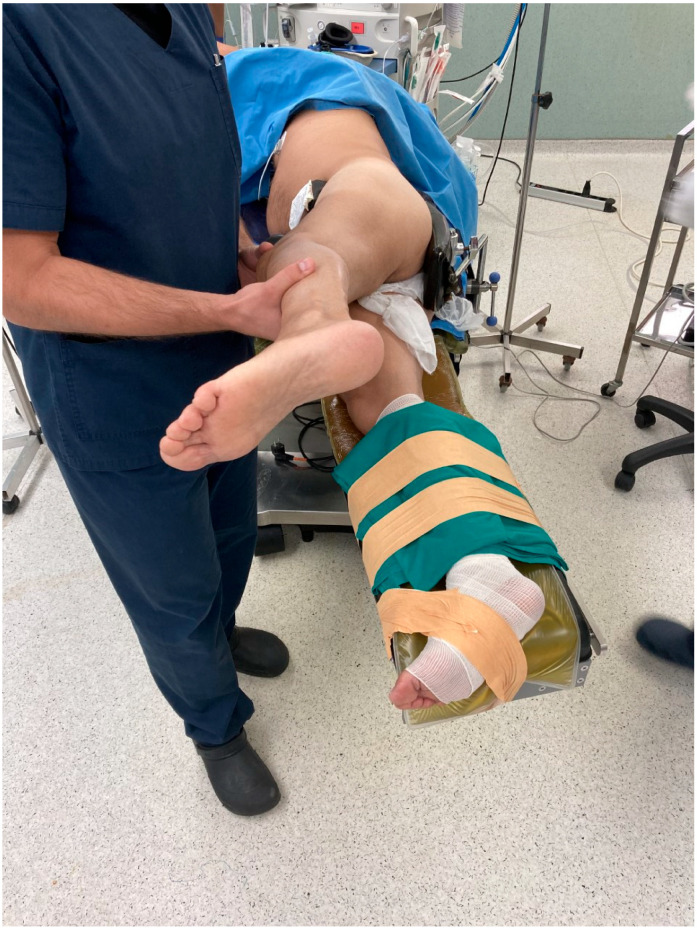
Positioning of the patient, the anterior leg support has been removed.

Under general, subarachnoid or epidural anesthesia, the patient is positioned in a lateral decubitus position, with the outer greater trochanter aligned to the surgical table’s edge. This allows for the removal of the front leg support. Simultaneously, the opposite leg is stabilized on the back leg support, which is extended approximately 20–30 degrees for improved visibility during femoral preparation (Figure 1).

**Figure 2:**
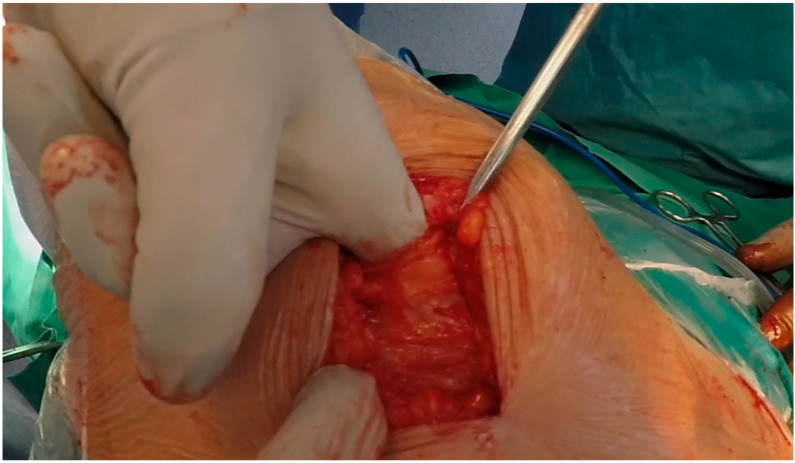
The main surgical step of the modified ALMIS approach; the interval between the gluteus medius and tensor fascia latae is exposed.

An approximately 8 cm longitudinal incision is made above the greater trochanter (the incision may extend with respect to the body weight and the specific anatomy of each patient). About 4 cm incisions are created both distally and proximally relative to the apex of the greater trochanter in a straight line defined by the greater trochanter and the lateral femoral condyle (longitudinal axis). The incision is centered at the midpoint of the greater trochanter. After splitting the tensor fasciae latae aponeurosis (towards the anterior iliac spine proximally and along the longitudinal femoral axis distally), the interval between the TFL, GM and vastus lateralis is exposed with, initially, a blunt Hohmann retractor and then a sharp curved one (Figure 2). The retractor is placed at the acetabulum roof or vertically at the femoral neck. The anterior 25% of the GM and the gluteus minimus are elevated. Following, capsulotomy, femoral head dislocation and head excision, Hohmann retractors are placed along the acetabular ring, while the limb is held in external rotation for better visualization. Capsular preparation, osteophyte resection and acetabular preparation are carried out using conventional techniques. The threaded cup is then placed (40–45° inclination and up to 10° anteversion).

During femoral exposure, the lower limb is positioned with less than 20 degrees of flexion, 20 degrees of adduction and 90 degrees of external rotation in front of the table, over the contra-lateral knee joint, at the location where the anterior leg support was removed. Curved rasps are used for the femoral preparation and a curved press-fit stem is placed. The gluteus minimus is reattached at its origin, while the anterior fibers of the GM are also reattached. Closure of the fascia latae, subcutaneous tissues and skin is performed using standard techniques.

The postoperative physiotherapy program is focused on promoting early mobilization, enhancing coordination, stability and strengthening of the hip muscles, particularly the abductors, to enhance range of motion and walking abilities. Physiotherapy was initiated immediately after the surgery, incorporating exercises for walking training (such as sit-to-stand, using crutches, and ascending/descending stairs) and bed exercises that included isometric quadriceps strengthening. During the initial 6 weeks, the use of walking aids was advised, and specific emphasis was placed on performing abductor exercises. Abductor exercises were performed initially in a supine position, while after the 1st week in a standing position. After the 4th postoperative week, gradual resistance was initiated.

## 3. Results

From the medical records a total of 688 patients had undergone THA with the ALMIS approach during the study period, while 603 of them (87.6%) could be located and re-evaluated for the final follow-up.

The studied population’s (603 patients) mean age was 69.4 years (standard deviation (SD) = 5.3), most of them were females (397; 65.8%), while mean BMI was 27.7 kg/m^2^ (SD = 1.9) The mean final follow-up of these cases was 6.9 years (SD = 0.9). From the medical records, at the 1-month follow-up, data were available for 512 patients, at the 3-month one for 476 and at the 12-month one for 577. At the final follow-up (mean 6.9 years), all these 603 patients were located and re-evaluated. The mean LHS of the studied population was 3.03 days (SD = 1.06).

The preoperative HHS had a median of 47 (with interquartile range (IQR) of 36 to 58). Following the surgery, at the 1-month follow-up the median HHS was 74 (IQR = 65 to 78), (*p*-value < 0.0001, compared to the preoperative HHS). At the 3-month follow-up, the median HHS was 93 (IQR = 89 to 96), and at the 12-month follow-up the median HHS was 95 (IQR = 90–99). At the final follow-up (mean 6.9 years), the median HHS was 94 (IQR = 87–100).

Table 1 highlights the range of motion (ROM) findings of the studied population at each follow-up time-point. Regarding the preoperative (baseline) ROM, the mean adduction was 15.4° (SD= 3.2), the mean abduction 18.2° (SD = 3.6), the mean flexion 79.1° (SD = 8), the mean external rotation 12.1° (SD = 3.6) and the mean internal rotation 7.2° (SD = 3.3). At the 1-month follow-up, the mean adduction was 19.9° (SD = 3.3; *p*-value < 0.0001 when compared to the baseline preoperative value), the mean abduction was 24.3° (SD = 3.1; *p*-value < 0.0001), the mean flexion 107.8° (SD = 9.1; *p*-value < 0.0001), the mean external rotation 20.1° (SD = 2.8; *p*-value < 0.0001) and the mean internal rotation 15.3° (SD = 2.7; *p*-value < 0.0001). At the 3-month follow-up, the mean adduction was 21.8° (SD = 3.6), the mean abduction 26.8° (SD = 3.5), the mean flexion 117.3° (SD = 11.1), the mean external rotation 23.4° (SD = 2.4) and the mean internal rotation 18.5° (SD = 3.7). At the 12-month follow-up, the mean adduction was 21.9° (SD = 3.5), the mean abduction 27° (SD = 3.9), the mean flexion 117.7° (SD = 10.7), the mean external rotation 24.3° (SD = 2.5) and the mean internal rotation 19.1° (SD = 3.1). At the final follow-up (mean 6.9 years), the mean adduction was 22° (SD = 3.4), the mean abduction 27.1° (SD = 4), the mean flexion 119.8° (SD = 9.9), the mean external rotation 24.4° (SD = 2.7) and the mean internal rotation 19.7° (SD = 2.7).

At the final follow-up, the mean incision length was 9.4 cm (SD = 1.2). Regarding complications, in eight patients (1.3%), anterior dislocation of the artificial joint occurred due to a traumatic event and they all underwent successfully close reduction. No revisions were performed in this patient group. Intraoperatively, in 11 patients (1.8%) a femoral fracture occurred during preparation of the femur. In three (27.3%) of these patients, a long press-fit stem was placed, in seven (63.6%) of them cerclages were placed, while one (9.1%) was treated conservatively. Periprosthetic infection was diagnosed in seven patients (1.2%). Three of them (42.9%) were treated with implant retention, polyethylene and femoral head exchange, debridement and antibiotics, since the infection was diagnosed during the early postoperative period (<3 weeks), while the remaining four (57.1%) were treated with two-stage revision arthroplasty. Two (0.3%) aseptic loosening cases of the acetabulum component were also revised in the study period.

## 4. Discussion

The ALMIS technique for THA was introduced in 2004 by Röttinger and in 2017 modified by Christodoulou [8,9]. More specifically, the positioning of the patient was changed, allowing for better visualization during preparation and the anterior ¼ of the GM was elevated, allowing easier access to the hip joint and the acetabulum. This technique has not been thoroughly investigated and limited data exist regrading tips and tricks and outcomes, as well as complications. This study re-described the technique and evaluated, retrospectively, 603 patients, from two centers, undergoing the ALMIS technique for THA.

Regarding the technique, it should be noted that no MIS instrumentation is necessary for the procedure, while all preparations and visualization of each anatomical region are carried out with standard tools and Hohmann retractors [9,10]. The positioning of the patient plays an important role, especially for the femoral preparation, while the external rotation of the lower limb during acetabular preparation enables adequate visualization.

Furthermore, it is important to locate the interval between the TFL, GM and vastus lateralis. Then, the elevation of the anterior ¼ of the GM fibers should be performed without expanding the incision to the vastus lateralis, hence limiting the muscular damage, as well as avoiding vascular injury and bleeding [12,13]. This surgical field typically poses no significant risk to vessels, making the approach relatively simple and bloodless. Additionally, during femoral head dislocation, in difficult cases, such as acetabular protrusion, a double osteotomy of the femoral neck may be performed. Placement of the cup component should be limited up to 10° anteversion, since in this approach the artificial joint is dislocated anteriorly. Through this preparation a press-fit or cemented cup may be placed as well.

In this modified ALMIS approach, the anterior fibers of the GM are elevated. It should be noted that the main gluteus muscle mass remains intact, while the anterior 1/3 contributes to internal rotation of the hip, as well as abduction [14,15,16]. Hence, theoretically, less Trendelenburg gait may be noticed, when compared to the direct lateral approach [15,16,17,18]. However, data about Trendelenburg were not available in this cohort.

Adequate training is of paramount importance for familiarizing with the approach. It has been documented that higher-level volume centers and surgeons yield better outcomes than lower-level ones [10,19,20]. Furthermore, although the ALMIS technique does not require MIS instrumentation, it should be noted that it requires two assistants who are familiarized with the approach.

Regarding evaluation of clinical outcomes of the ALMIS technique, the HHS was used. The HHS is a clinical assessment tool used to evaluate the functional outcome and measure the severity of hip-related conditions or injuries. It was developed by Dr. William H. Harris in 1969 and has since become widely used in orthopedics and hip surgery [11]. The HHS consists of a set of questions and physical examinations that assess various aspects of hip function and pain. It covers a range of parameters, including pain level, walking ability, range of motion, and activities of daily living. The final HHS ranges from 0 to 100, with a higher score indicating better hip function and less pain. The interpretation of the score is as follows: Excellent: 90–100 points, Good: 80–89 points, Fair: 70–79 points, Poor: <70 points [11]. The mean HHS of this cohort had already improved significantly at the 1-month follow-up, exhibiting quick improvement that could be attributed to the MIS, while the mean score was in the excellent outcomes group. Similar results have been reported from other smaller cohorts regarding the ALMIS technique, as well as other minimally invasive approaches for THA [21,22,23].

ROM of the hip joint is an under-investigated parameter. Many clinical scores encompass some data regarding ROM but thorough evaluation of all hip movements is not frequently reported [24,25]. All hip movements, including adduction. abduction, flexion and external and internal rotation showed significant improvement at the 1-month follow-up. This parameter also reveals quick rehabilitation and clinical improvement.

In this study, threaded cups were used. The 3rd generation threaded cups have revealed high primary stability, achieved with helicoids, representing an essential factor for good subsequent osteointegration and, hence, also for long-term secondary stability [21,26,27,28,29]. Although threaded cups have revealed biomechanical advantages when compared to press-fit cups, they are not frequently used [26,29]. This could be attributed to the failure of the 1st generation implants, which by having a smooth surface led to high rates of implant breakage and loosening [21]. These problems no longer exist with the 3rd generation implants that have a porous-coated surface [28,29]. Nevertheless, they have not yet been established as the gold standard. Although the purpose of this study was not to evaluate the implants used, in this cohort (603 patients), aseptic loosening was reported only in 0.3% during a mean 6.9-year follow-up. It should be noted that this may be under-estimated, since the patients that could not be located for the final follow-up were not included in the study. Therefore, it could be possible that most of the revision cases could be in the group that was excluded.

Regarding complications, the intraoperative femoral fractures could be considered approach-related. Their incidence in the present cohort was 1.8%. It has been documented that the incidence of intraoperative fractures of the femur is higher in the anterior, anterolateral and lateral approaches in comparison to the posterior approach [30]. Minimally invasive techniques may also negatively contribute to such adverse effects. In the modified ALMIS technique, to avoid intraoperative femoral fractures, the opposite leg is stabilized on the back leg support, which is extended approximately 20–30 degrees, while during preparation the operation table may be titled 20–30 degrees, allowing for better visualization. In difficult cases, the incision may also be extended. Furthermore, the rate of periprosthetic joint infection, requiring further surgery, was 1.2% in this cohort. The incidence of hip periprosthetic infection is estimated to be between 1 and 2% [31].

The present study has some limitations. It is a retrospective study that does not encompass a control group and comparison with other minimally invasive approaches was not possible. At each follow-up (1 month, 3 months, 12 months), there were not data for all patients or for early postoperative pain scores and operation time, which is attributed to the retrospective nature of the study. Nevertheless, this study represents the largest cohort of patients undergoing the modified ALMIS technique for THA with threaded cups, with a mean 6.9-year follow-up, providing useful insights regarding the technique, as well as the patients’ outcomes.

## 5. Conclusions

This modified ALMIS technique is a minimal invasive approach, providing good visibility during acetabular and femoral preparation, while no specific MIS instrumentation is required. The modified ALMIS technique revealed excellent clinical outcomes at short-, mid- and long-term follow-up, by significantly improving hip range of motion and the HHS. Careful utilization of this technique, after adequate training, should yield favorable outcomes, while relatively minimal major complications, such as intraoperative femoral fractures or periprosthetic infections, should be expected.

## Figures and Tables

**Table 1:** Detailed data regarding range of hip motion of the studied population.

Range of Motion (ROM)					
	Preop N = 603	1 Month N = 512	3 Months N = 476	1 Year N = 577	6.9 Years (Final Follow-up) N = 603
Mean adduction (°)	15.4 ± 3.2 (7–25)	19.9 ± 3.3 (15–25)	21.8 ± 3.6 (15–30)	21.9 ± 3.5 (15–20)	22.0 ± 3.4 (15–30)
Mean change compared to the baseline (°)	-	4.5	6.4	6.5	6.6
*p*-value compared to baseline	-	<0.0001 *	<0.0001 *	<0.001 *	<0.0001 *
Mean abduction (°)	18.2 ± 3.6 (10–30)	24.3 ± 3.1 (15–30)	26.8 ± 3.5 (20–40)	27 ± 3.9 (20–35)	27.1 ± 4.0 (20–40)
Mean change compared to the baseline (°)	-	6.1	6.8	8.8	8.9
*p*-value compared to baseline	-	<0.0001 *	<0.0001 *	<0.0001 *	<0.0001 *
Mean flexion (°)	79.1 ± 8.0 (60–100)	107.8 ± 9.1 (80–125)	117.3 ± 11.1 (80–125)	117.7 ± 10.7 (80–130)	119.8 ± 9.9 (85–135)
Mean change compared to the baseline (°)	-	28.7	38.2	38.6	40.7
*p*-value compared to baseline	-	<0.0001 *	<0.0001 *	<0.0001 *	<0.0001 *
Mean external rotation (°)	12.1 ± 3.6 (0–20)	20.1 ± 2.8 (15–25)	23.4 ± 2.4 (20–25)	24.3 ± 2.5 (20–30)	24.4 ± 2.7 (20–35)
Mean change compared to the baseline (°)	-	8	11.3	12.2	12.3
*p*-value compared to baseline	-	<0.0001 *	<0.0001 *	<0.0001 *	<0.0001 *
Mean internal rotation (°)	7.2 ± 3.3 (0–15)	15.3 ± 2.7 (10–25)	18.5 ± 2.7 (10–25)	19.1 ± 3.1 (10–25)	19.7 ± 2.7 (10–25)
Mean change compared to the baseline (°)	-	8.1	11.3	11.9	12.5
*p*-value compared to baseline	-	<0.0001 *	<0.0001 *	<0.0001 *	<0.0001 *

(*): statistically significant.

## Data Availability

Research data is available after reasonable request with the corresponding author.
